# Impact of the 2023 ACR/EULAR Classification Criteria in Women with Primary Antiphospholipid Syndrome during Pregnancy

**DOI:** 10.3390/diagnostics14192162

**Published:** 2024-09-28

**Authors:** Víctor M. Martínez-Taboada, Ana Micieces Gómez, Sara del Barrio-Longarela, Ana Merino, Alejandra Comins-Boo, Marcos López-Hoyos, Leyre Riancho-Zarrabeitia, Rafael Gálvez, José L. Hernández

**Affiliations:** 1 Division of Rheumatology, Hospital Marqués de Valdecilla-IDIVAL, 39008 Santander, Spain; ana.micieces@alumnos.unican.es (A.M.G.); rafael.galvez@scsalud.es (R.G.); 2 Departamento de Medicina y Psiquiatría, Universidad de Cantabria, 39005 Santander, Spain; hernandezjluis@gmail.com; 3 Division of Obstetrics and Gynecology, Hospital Marqués de Valdecilla, 39008 Santander, Spain; saradelbarrio@hotmail.com (S.d.B.-L.); anaisabelmerino@gmail.com (A.M.); 4 Immunology Department, Hospital Universitario Marqués de Valdecilla-IDIVAL, 39011 Santander, Spain; alejandra.comins@scsalud.es (A.C.-B.); marcos.lopez@scsalud.es (M.L.-H.); 5 Departamento de Biología Molecular, Universidad de Cantabria, 39005 Santander, Spain; 6 Rheumatology Department, Hospital Sierrallana-IDIVAL, 39300 Torrelavega, Spain; leyre.riancho@scsalud.es; 7 Department of Internal Medicine, Hospital Marqués de Valdecilla-IDIVAL, 39008 Santander, Spain

**Keywords:** pregnancy, obstetric morbidity, fetal loss, antiphospholipid syndrome, antiphospholipid antibodies, classification criteria

## Abstract

**Background/Objectives**: ACR/EULAR has recently developed new classification criteria for antiphospholipid syndrome (APS). The present study aims to analyze the impact of these new 2023 ACR/EULAR classification criteria in a cohort of pregnant women with primary APS. **Methods**: Retrospective cohort study of 93 consecutive pregnant women attending the Autoimmune Diseases Pregnancy Clinic, a multidisciplinary unit of a tertiary care teaching hospital, between 2005 and 2023. All of them fulfilled the Sydney classification criteria for APS. Women diagnosed with rheumatic autoimmune diseases other than APS were excluded. **Results**: Twenty-four out of ninety-three patients (25.8%) met the 2023 ACR/EULAR criteria for APS. Patients who met the new classification criteria were very similar to those who did not, except for being younger (*p* < 0.001), and had a lower number of clinical pregnancies (*p* = 0.004). The obstetric domain was clearly underrepresented in women who fulfilled the 2023 ACR/EULAR criteria (*p* < 0.001). Patients meeting the new classification criteria were primarily characterized by preterm births before 34 weeks due to severe placentation disorders (*p* = 0.004). Women with early and late fetal loss were significantly underrepresented (*p* < 0.0001 and 0.03, respectively). Nearly half of these patients had thrombocytopenia (*p* < 0.001). Serologically, these patients showed a higher frequency of persistent lupus anticoagulant (*p* = 0.02) and a lower frequency of IgM isotype antiphospholipid antibodies (*p* = 0.05). **Conclusions**: Almost three-quarters of the patients included in the study did not meet the 2023 ACR/EULAR criteria. Most patients who could not be classified according to these new classification criteria were those with early and/or late fetal deaths, as well as patients carrying only IgM aCL/AB2GPI antibodies. The high specificity of the 2023 ACR/EULAR criteria, restricted to severe placentation disorders, may leave the majority of patients with obstetric APS out of the new classification criteria.

## 1. Introduction

Antiphospholipid syndrome (APS) is an autoimmune disease characterized by thrombotic and/or obstetric events, associated with the presence of persistent antiphospholipid antibodies (aPL) [1]. As in all autoimmune diseases, the scientific community, represented by experts in this field, has developed classification criteria in an attempt to standardize research studies at both the clinical and basic level [1]. Regarding APS, until the end of 2023, physicians have been using the Sapporo classification criteria [2], later revised in Sydney [1]. These criteria allowed a patient to be classified as APS if they met at least one clinical and one serological criterion. More recently, the ACR/EULAR, in the same way as previously done with most rheumatic diseases, has developed new classification criteria that will undoubtedly change the perspective of the disease [3]. In addition to the important advances in the methodology (international committee of experts, rigorous definitions, international case capture, inclusion of two validation cohorts, and a powerful statistical analysis), the main objective has been to develop highly specific criteria to identify homogeneous groups of patients, which allows progress in APS research [3,4]. From a practical point of view, and independently of the commented methodological issues, these new criteria aimed to improve the limitations of the previous ones [5]. In this sense, from a clinical point of view, the previous criteria did not include risk factors for thrombosis and less frequent manifestations such as valvular disease, thrombocytopenia, or the involvement of microvasculature. Furthermore, the definition of obstetric morbidity was perhaps too general and included a frequent, but unspecific, clinical manifestation, such as recurrent early pregnancy loss. On the other hand, and from a laboratory point of view, the stratification of aPL and a clearer definition of their positivity were also part of the weakness of Sydney criteria [5].

The interest in these new classification criteria has prompted the rapid publication of a non-negligible number of studies [6,7,8,9,10,11], several of them in a “letter to the editor” format [6,7,8], about the impact of these new criteria. Although most of them are indeed aimed at analyzing the global sensitivity and specificity of the new criteria, most have been carried out in populations of Asian origin [6,7,8,10] and include the full spectrum of the disease. Only one study carried out in Europe has analyzed the impact of the new classification criteria in patients with APS [9]. Most studies indeed agree on the high specificity of the new criteria [6,7,8,10,11], but they all show a clear decrease in their sensitivity that, from a clinical point of view, is basically linked to the obstetric domain [6,7,8,9,10,11]. On the other hand, all of the studies published to date include a significant proportion of patients with APS associated with other autoimmune diseases, which makes it difficult to interpret some of the new clinical manifestations included in the 2023 EULAR/ACR criteria, such as thrombocytopenia.

Taking into account these considerations, our study aimed to analyze the impact of the new 2023 ACR/EULAR classification criteria in a cohort of pregnant women from a single center and without other associated systemic autoimmune diseases.

## 2. Materials and Methods

### 2.1. Study Participants

This retrospective cohort study included 93 consecutive pregnant women followed at the Autoimmune Diseases Pregnancy Clinic, a multidisciplinary unit of a tertiary care teaching hospital, between 2005 and 2023. All of them were classified according to the Sydney classification criteria [1]. Women who fulfilled the classification criteria for rheumatic autoimmune diseases other than APS were excluded. The information collected from individual cases was completely anonymized, and the study was approved by the Ethics Committee of Cantabria (internal code: 2024.043).

### 2.2. Data Collection

Data were collected using a prespecified standardized questionnaire in a computerized database. We assessed the following clinical variables:

- Demographic and general characteristics: age, sex, body mass index (BMI), current/past tobacco use, high blood pressure (equal or greater than 140/90 mm Hg or being on antihypertensive agents), dyslipidemia (serum total cholesterol or triglyceride levels greater than 230 mg/dl and 150 mg/dl, respectively, or being on lipid-lowering drugs), diabetes mellitus (according to the ADA criteria), and past or present family (<50 years) or personal history of thrombotic disease.
- Comorbidities: the three main entities associated with pregnancy outcomes were also recorded: (a) inherited thrombophilia (factor V Leiden, prothrombin mutation, protein S, and/or protein C deficiency); (b) thyroid disease (history of hypo/hyperthyroidism or the presence of confirmed specific autoantibodies); and (c) obstetric comorbidity (local uterine abnormalities, endometriosis, and polycystic ovary syndrome).

### 2.3. Clinical Domains

Clinical manifestations were collected according to the Sydney definitions [1] and 2023 ACR/EULAR domains [3]. Clinical manifestations according to the Sydney definitions in this cohort had been previously reported [12,13]. New clinical 2023 ACR/EULAR domains, including macrovascular (domains 1 and 2), microvascular (domain 3), obstetric (domain 4), cardiac valve (domain 5), and thrombocytopenia (domain 6), were retrospectively recorded according to the strict proposed definitions [3].

### 2.4. Autoantibody Assessment and Serological Domains

The presence of the following antibodies and aPL isotypes was quantified by commercial enzyme immunoassay in a solid phase (ELISA; OrgentecDiagnostika GmbH, Mainz, Germany): anticardiolipin antibodies (aCL) and anti-beta2 glycoprotein I antibodies (AB2GPI) of the IgG and IgM isotypes. The results are reported as quantitative and semiquantitative values. Thus, aCL are quantified in GPL (aCL IgG) or MPL (aCL IgM) according to the standard curve constructed in each test with 5 dilution points of the Harris/Sapporo standards. AB2GPI are quantified as U/mL. Only medium-high titers of aPL were considered positive. The criteria recommended by the International Society of Thrombosis and Hemostasis (ISTH) Scientific and Standardization Committee (ISTH) for the standardization of lupus anticoagulant/antiphospholipid antibodies (LA/APA) were applied for the characterization of LA [14,15,16]. New serological ACR/EULAR domains, including LA (domain 7) and aCL/AB2GPI (domain 8), were also retrospectively recorded [3].

### 2.5. Pregnancy Morbidity Definitions

* Obstetric manifestations: (a) Sydney criteria [1]; (b) non-criteria obstetric morbidity related to APS: 1–2 early pregnancy losses (<10 weeks), preterm birth (between 34 and 36 + 6 weeks), late preeclampsia (>34 weeks), and abruptio placentae and unexplained in vitro fertilization failures (>2) [17].
* Pregnancy loss: early pregnancy loss (<10 weeks) and/or fetal death (>10 weeks).
* Adverse pregnancy outcome (APO): early pregnancy loss, fetal death, preeclampsia, abruptio placentae, and preterm birth (<37 weeks).

### 2.6. Statistical Analysis

Quantitative variables were expressed as the mean ± standard deviation (SD) or median [interquartile range] and categorical variables in percentage. Student’s *t*-test, Mann–Whitney *U* test, and ANOVA were used to compare quantitative variables and Pearson’s chi-square test in the case of categorical variables. All tests were two-tailed, and significance was set at *p* < 0.05. Analyses were conducted using the SPSS 29.0 statistical package (IBM Corporation, New York, NY, USA).

## 3. Results

### 3.1. General Features of the Study Cohort

During the study period, 93 consecutive patients fulfilled the inclusion criteria. The main baseline characteristics of the cohort (Table 1), clinical manifestations according to Sydney criteria, obstetric morbidity (Table 2), and standard of care (SoC) treatment (Table 3) are shown. The mean age of the overall group was 34 ± 5.5 years, and the patients were followed up for 60 (18–159) months. There was a median of four [3.0–5.0] pregnancies per patient. Overall, and despite being a population of women of childbearing age, the prevalence of cardiovascular risk factors was very high (64.5%). The more common comorbidities with a potential impact on the obstetric outcome were also frequent (Table 1).

**Figure 1:**
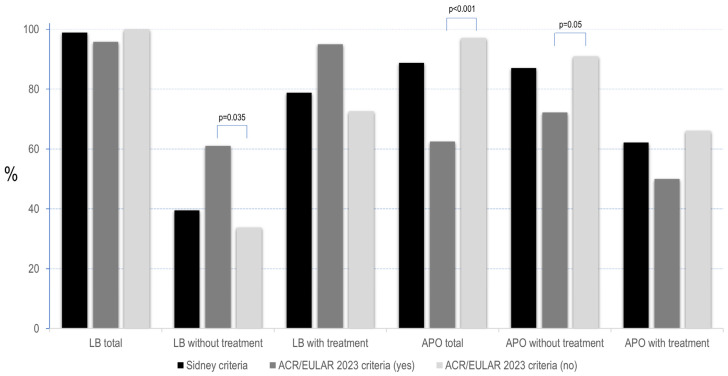
Main pregnancy outcomes according to the Sidney and the 2023 ACR/EULAR classification criteria. The figure shows the main pregnancy outcomes, including live birth (LB) and adverse pregnancy outcomes (APO), with and without treatment, in patients who fulfilled the Sidney and the 2023 ACR/EULAR classification criteria.

From a serological point of view, 54.8% of the patients had a high-risk serologic phenotype, including double/triple positivity or the isolated presence of LA (Table 4). After diagnosis, most of them received SoC treatment with LDA and/or LMWH during pregnancy [18,19,20,21] (Table 3). As shown in Figure 1 and Supplementary Table S1, the addition of SoC treatment significantly improved the obstetric outcome, both by achieving a live birth and decreasing APO. Eighteen out of ninety-three patients who attended the pregnancy clinic had previously presented a thrombotic episode, and only one of them simultaneously met the Sydney obstetric clinical criteria. The rest of the patients had only obstetric classification criteria manifestations [2].

### 3.2. How Many Patients Classified as APS According to the Sydney Criteria Meet the 2023 ACR/EULAR Criteria?

**Figure 2:**
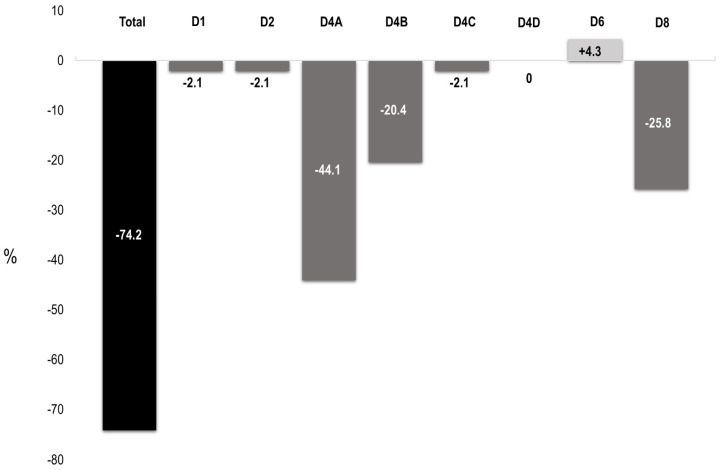
Impact of the 2023 ACR/EULAR classification criteria in patients with antiphospholipid syndrome followed during pregnancy. The figure shows the overall impact and the impact according to the different domains (D) included in the new classification criteria. D4A: ≥3 pre-fetal abortion (<10 weeks) and/or fetal (10 w 0 d–15 w 6 d) deaths; D4B: fetal (16 w 0 d–33 w 6 d) deaths in the absence of PEC or PI; D4C: preeclampsia with severe features (<34 w 0 d) or PI with severe features (<34 w 0 d) with/without fetal death; D4D: preeclampsia with severe features (<34 w 0 d) and PI with severe features (<34 w 0 d) with/without fetal death.

As shown in Figure 2, 24 out of 93 patients (25.8%) included in the study met the 2023 ACR/EULAR criteria. The majority of patients who could not be classified according to the new classification criteria were those with early and/or late fetal deaths (Table 5), as well as patients carrying only IgM aCL/AB2GPI antibodies (Table 4). Only a minority of patients with thrombotic phenomena, with risk factors for thrombosis, or patients with preterm delivery <34 weeks who did not meet the strict severity criteria for preeclampsia and/or placental insufficiency, no longer met the new criteria. It should be pointed out that none of the patients included in the study presented clinical manifestations included in the new domains (domains 3 and 5), except thrombocytopenia (domain 6). Overall, 13% of patients presented thrombocytopenia, but only in 4.3% of cases did this domain allow the classification of patients according to the 2023 ACR/EULAR criteria.

### 3.3. How Different Are Patients Who Meet the 2023 ACR/EULAR Criteria?

According to the general characteristics, the patients who met the new classification criteria were very similar to those who did not, except for being younger (*p* < 0.001) and presenting a lower frequency of thyroid disease (*p* = 0.06) (Table 1). Furthermore, patients fulfilling the new criteria had a lower number of clinical pregnancies (*p* = 0.004).

The new classification criteria also determined a clear differential clinical profile. On the one hand, thrombotic domains (domain 1: *p* < 0.001 and domain 2: *p* = 0.037) were more prevalent in those patients who met the new criteria, mainly due to the lack of high-risk thrombotic factors in the study population (Table 5). From a purely obstetric point of view, the obstetric domain was clearly underrepresented in those patients who fulfilled the 2023 ACR/EULAR criteria (*p* < 0.001). Patients included in the new criteria were mainly characterized by the presence of preterm births <34 weeks, due to placentation disorders with severity criteria (*p* = 0.004), the subgroup of women with early and late fetal loss being clearly underrepresented (*p* < 0.0001 and 0.03, respectively) (Table 5). Finally, almost half of the patients who met the new classification criteria presented thrombocytopenia (*p* < 0.001).

From a serological point of view, patients who meet the new criteria have a higher frequency of persistent LA (*p* = 0.02) and a clear tendency to present IgM isotype aPL less frequently (*p* = 0.05). Patients who met the new classification criteria tended to present a high-risk serological profile more frequently (*p* = 0.06).

Although we found no significant differences regarding treatment, patients who met the new classification criteria more frequently received LMWH (*p* = 0.05) or in combination with LDA (*p* = 0.09) (Table 3).

Virtually all patients had at least one live newborn during follow-up, and we found no overall differences between patients who did or did not meet the new classification criteria (Figure 1 and Supplementary Table S1). However, patients who fulfilled them had a significantly higher rate of live births without treatment (61% vs. 33.8%, *p* = 0.035). This difference disappeared with treatment, but a trend persisted in the group of patients who met the new criteria (95% vs. 72.6%, *p* = 0.06) (Supplementary Table S1). Patients who did not fulfill the 2023 ACR/EULAR had overall more APO (62.5% vs. 97.1, *p* < 0.001), especially without treatment (72.2% vs. 91%, *p* = 0.05), although the results were similar after SoC therapy (Figure 1 and Supplementary Table S1). Noteworthy, when each APO was analyzed individually, both without treatment and after treatment, all of them improved, except for the development of preeclampsia (*p* = 0.013) in the group of patients who met the new classification criteria (Table 6).

## 4. Discussion

After the publication of the new ACR/EULAR classification criteria in 2023 [3], a relevant number of studies have already been published aimed at validating their specificity and sensitivity in cohorts of patients with suspected APS [6,7,8,9,10,11]. Nevertheless, our study is the first to evaluate its impact in a cohort of pregnant patients with APS without other related autoimmune diseases.

Our results, such as previous ones, highlight that the new classification criteria, despite their high specificity, are accompanied by a low sensitivity due to their impact on the obstetric domain. The application of these new criteria in women of fertile age, mainly with obstetric manifestations, clearly defines a clinical subgroup characterized by placentation disorders with severity features. Whether this high sensitivity is going to be especially useful in advancing knowledge on the pathogenesis or the development of new therapeutic alternatives in this very specific subgroup of patients will have to be tested in new cohorts of patients and properly designed prospective multicenter studies.

From a general point of view, both groups of patients, those who meet the new criteria and those who only meet the Sidney criteria, are very similar. However, there are two relevant and probably linked aspects that differentiate them. Firstly, patients who meet the 2023 ACR/EULAR criteria are younger and, secondly, had a significantly lower number of pregnancies. One can argue that the subgroup of patients who meet the new criteria has a more severe disease (thrombotic phenomena and/or placentation disorders) and debuts at an earlier age, while those patients who only meet the Sidney criteria present a very different clinical phenotype, where recurrent fetal loss is the main manifestation. While the majority of women who currently develop serious placentation disorders can achieve a live newborn, those women with fetal losses delay their motherhood due to the accumulation of APO. An alternative explanation could be that those women with more severe manifestations of the disease decide or are advised to have fewer pregnancies.

Despite the clinical differences between both subgroups of patients, the final obstetric outcome was similar with a very high live birth rate. Furthermore, as expected, we did not find statistically significant differences in terms of the therapeutic scheme. When we analyzed the APO globally, the group of patients who only met the Sidney criteria had a higher frequency, attributed to early fetal losses, which was corrected with the SoC treatment. However, preeclampsia, which was significantly more frequent in patients who met the 2023 ACR/EULAR criteria, continues to be so, despite treatment. In this regard, it is well known that one of the most relevant risk factors for the development of preeclampsia is having had one or several previous episodes [22]. Moreover, the specific treatment of this manifestation in patients with APS has not been well established [23].

From a serological point of view, the only significant difference between both groups of patients was the persistent positivity for LA. However, although the difference did not reach statistical significance (*p* = 0.05), positivity for IgM antibodies was the one that had the greatest impact on the final classification of patients according to the new criteria. In fact, one-quarter of the patients excluded from the new classification criteria had only IgM isotype aPL. It has been suggested that the presence of IgM antibodies is less specific for APS [24,25,26,27], but there are controversies on this issue, especially in patients who express persistently high titers and even double positive for aCL and AB2GPI [28,29,30,31,32].

Classification criteria are primarily intended to create well-defined and relatively homogenous cohorts for clinical research that capture the majority of patients with key shared features of a certain disease [33]. Validated classification criteria are considered critical to the interpretation of study findings and comparisons of results between different studies. Classification criteria should have high specificity, which generally comes at the expense of somewhat lower sensitivity. Consequently, few individuals are incorrectly labeled as having a disease (false positives), but a proportion of individuals with the disease diagnosis may be missed (false negatives). This may make classification criteria inappropriate for use in routine clinical care [33]. Thus, this may be the case for domain 4 (obstetric morbidity) of the new 2023 ACR/EULAR classification criteria. As we have shown, almost three-quarters of patients classified as APS according to the previous Sidney criteria cannot be classified with this new modality. In most cases, this discrepancy is due to the lower weight given to fetal deaths but with a clear influence also of the lower weight attributed to IgM aPL antibodies.

Although, indeed, fetal deaths not associated with placentation disorders are clearly less specific [34], they represent one of the most frequent clinical manifestations of APS [24,25,28,35,36]. Furthermore, it is striking that new domains such as the microvascular, with a very low or exceptional real frequency in some manifestations, have a higher classification weight than the most frequent manifestations of the obstetric domain. On the other hand, laboratory manifestations such as thrombocytopenia, which is not uncommon in patients with APS [35,37] but which is not accompanied by severity and does not require specific treatment in most cases, also obtain a high weight in the disease classification criteria. Finally, in patients without another associated connective tissue disease, preferably systemic lupus erythematosus (SLE), manifestations of the microvascular and valvular domain are usually infrequent [7]. This point added to the intrinsic difficulty of attributing a specific manifestation, such as thrombocytopenia or pulmonary hemorrhage, to APS and not to an associated connective tissue disease, the classification of patients being difficult [3,7].

As the Steering Committee of the new 2023 ACR/EULAR criteria recognizes in the discussion [3], they identified “controversial” clinical scenarios that we have highlighted in the present study (a) otherwise unexplained three or more consecutive pre-fetal deaths (<10 weeks) and/or early fetal death (10 weeks 0 days to 15 weeks 6 days) alone, with laboratory criteria score ≥3, (b) unexplained one or more fetal death (16 weeks 0 days to 34 weeks 0 days) alone, with laboratory criteria score ≥3, and finally, (c) moderate- or high-titer IgM aCL/anti-β2GPI antibodies with a clinical criteria score ≥3.

Overall, considering only severe placentation disorders with sufficient weight, which can represent approximately 10–15% of patients with APS and where prevention and treatment measures can correct a significant percentage of cases, can represent a dramatic restriction in the advance in knowledge on obstetric APS. Thus, a potential rectification allocating at least two points for recurrent miscarriages and fetal losses and allowing an independent scoring for both has been suggested [36]. Furthermore, in domain 8, the new criteria assign one point for persistent moderate-high IgM aCL/AB2GPI antibodies positivity, equating this with a single-time LA positivity. Valuable literature substantiates the association between persistent moderate-high titers of aCL/AB2GPI IgM isotypes in women with adverse obstetric outcomes [28,29,30]. However, an isolated single-time positive result for LA possesses limited, if any, established pathogenic potential [36]. Interestingly enough, aCL/AB2GPI IgM isotypes are used in risk scores for thrombosis, such as the aGAPSS score [38] or the EUREKA algorithm, to predict the risk of obstetric complications [31]. Therefore, assigning the same laboratory value to a solitary LA positivity as to persistent aCL/aβ2GPI IgM positivity also appears quite unbalanced [36].

The Steering Committee deemed it acceptable to exclude the majority of patients with fetal death without severe placentation disorders from the current APS classification but to further study them independently. The problem in this situation is what type of research is going to be carried out and what funding is going to be required, having excluded the most frequent manifestations within the obstetric morbidity spectrum. In this regard, it has been suggested that one of the possible advantages of the new classification criteria could be, given their high specificity, the ability to develop clinical trials that include a more homogeneous population of patients and that allow drawing more valid conclusions [9]. However, it seems reasonable to think that, in the case of manifestations as infrequent as those of the microvascular sphere or manifestations with doubtful clinical significance such as cardiac valvular involvement or thrombocytopenia, this advantage will be clearly diluted. Furthermore, in obstetric APS, most randomized controlled trials compare different thromboprophylaxis regimens and, to a lesser extent, the impact of corticosteroids, intravenous immunoglobulins, or hydroxychloroquine [39,40]. Noteworthy, all clinical trials, except one conducted in patients with recurrent early-onset preeclampsia [23], have predominantly included patients with recurrent early miscarriages [41]. Interestingly, the only study that included women with previous preterm birth <34 weeks (secondary to placental disorder) concluded with the enrollment of only 32 patients after 9 years of recruitment across 17 centers [23]. Moreover, it is important to highlight that, despite current recommendations in this clinical context [19,40], this study did not demonstrate the superiority of the combined treatment (LMWH plus LDA) over LDA monotherapy [23].

Our study has certain limitations. First of all, those inherent to a retrospective design. In addition, it is carried out in a single center and a multidisciplinary unit specifically devoted to the treatment of obstetric complications in patients with autoimmune diseases. This means that the results cannot be extrapolated to other populations and probably to the care of pregnant patients outside specialized units. Another possible limitation is that the present study includes all patients with APS (both thrombotic and obstetric) followed during pregnancy. It can be speculated that the inclusion of patients from the thrombotic subgroup could influence our results. However, once excluding patients with pure thrombotic APS, we obtained very similar results to the overall analysis. Finally, the analysis of the patients, and not of each of the pregnancies separately, does not allow us to determine with certainty the impact of the treatment in each of the groups or the proportion of patients who could meet the criteria during the follow-up. While the first aspect is being analyzed, the second is unlikely to impact the results, since the only clinical manifestation that does not improve significantly with medical treatment is preeclampsia, and this mainly occurs in the group of patients who meet the new clinical criteria.

We consider that our study has several advantages over previous ones. Firstly, these studies have been carried out in patients with aPL associated with other autoimmune diseases, mainly SLE [6,7,8,9], whereas those patients have been excluded from our study. Thus, we could analyze a more homogeneous population of patients belonging to the clinical spectrum of primary APS. Secondly, the present cohort represents the whole spectrum of patients with APS followed during pregnancy and ranges from pure thrombotic to pure obstetric APS. Finally, all patients have been followed and treated during their pregnancies by the same group of physicians, which avoids heterogeneity in their management.

As has been previously pointed out [36], and following the impressive results obtained in our study and its great impact on patients with obstetric APS, it seems appropriate to suggest some proposals that could contribute to mitigating the impact on this very relevant group of patients. Although the scoring method includes the choice of the highest score within each domain, this aspect could be questionable in the obstetric domain, since the different clinical manifestations included are possibly due to different pathogenic mechanisms. In this sense, the pathogenic mechanisms of early abortion or early fetal death are different from those responsible for late fetal death or severe preeclampsia. For this reason, the possibility of adding scores on different clinical manifestations that often occur concurrently in the same patient (for example: three or more early abortions and a fetal death at 26 weeks) and that are highly suggestive of obstetric APS could contribute to classifying patients more accurately. Manifestations such as late fetal death should certainly have a score weight greater than 1. On the other hand, although the appearance of severe placental disorders before week 34 is probably more specific to the disease, a significant proportion of patients develop these complications between weeks 34 and 37. Consider these patients, especially if they have previously presented earlier complications (for example, recurrent abortions or early fetal death), it would allow a non-negligible number of patients to be properly classified. The other aspect that clearly impacts the classification of patients is the aCL/AB2GPI IgM antibody score. Although some authors may question the role of IgM antibodies, there is sufficient evidence about their possible relevant role in the development of obstetric manifestations, especially when its positivity is persistent and at moderate-high titers [28,29,30]. As has been done in domain 8 with the IgG isotype, a score according to the degree of positivity (for example, moderate: 1 point, and high: 2 points) and the antibody load (for example, double-positive high titer: 3 points) seems reasonable for IgM aPL carriers and would allow us to avoid excluding a significant percentage of patients. Most physicians with experience in taking care of pregnant women with APS would agree that a positive serology for aCL and AB2GPI IgM at a high titer is highly suggestive of the disease in the appropriate clinical context. Obviously, these proposals are only intended to improve the classification of an important subgroup of patients with obstetric APS and should be adequately tested.

In summary, the lower sensitivity of the 2023 ACR/EULAR criteria compared to the 2006 Sydney criteria could be attributed to the strict definition of pregnancy morbidity and the lower weight given to isolated IgM isotype aPLs. Nevertheless, the implementation of the new classification criteria should not prevent patients with APS during pregnancy from receiving appropriate treatment according to their main clinical manifestations and the current evidence available. Our results confirm the profound impact of these new classification criteria on one of the most frequent clinical manifestations of APS, such as obstetric morbidity, and highlight the potential difficulties in performing clinical studies in patients with the former “obstetric APS” if these criteria are fully applied in everyday practice.

## Figures and Tables

**Table 1:** Demographic characteristics, cardiovascular risk factors, and main comorbidities in patients fulfilling the Sidney and 2023 ACR/EULAR classification criteria.

	Sidney Criteria *N* = 93	2023 ACR/EULAR Criteria		
		YES *N* = 24	NO *N* = 69	*p*
Age, (years, mean ± SD	34 ± 5.5	30.9 ± 4.3	35.1 ± 5.5	<0.001
Time to diagnosis (m), median [IQR]	29.5 [13.3–56.8]	21.5 [4.5–54.3]	32 [14–56.8]	0.34
Follow-up (m), median [IQR]	60 [18–159]	78 [44.8–133.8]	47 [16.5–163.5]	0.21
Cardiovascular risk factors, *n* (%)	60 (64.5)	16 (66.7)	44/63.8)	0.79
- Obesity	22 (22.7)	7 (35)	15 (18.5)	0.36
- Smoking	41 (44.1)	11 (45.8)	30 (43.5)	0.84
- High blood pressure	7 (7.5)	1 (4.2)	6 (8.7)	0.67
- Diabetes	2 (2.2)	-	2 (2.9)	0.99
- Dyslipidemia	8 (8.6)	2 (8.3)	6 (8.7)	0.99
Comorbidities, *n* (%)				
- Hereditary thrombophilia	10 (12.5)	4 (17.4)	6 (10.5)	0.46
- Thyroid disease	11 (11.8)	-	11 (15.9)	0.06
- Obstetric comorbidity	9 (9.7)	3 (12.5)	6 (8.7)	0.69

SD: standard deviation; IQR: interquartile range.

**Table 2:** Clinical APS subgroups according to the Sydney criteria and obstetric morbidity related to APS in the different study groups.

	Sidney Criteria *N* = 93	2023 ACR/EULAR Criteria		
		YES *N* = 24	NO *N* = 69	*p*
Number of pregnancies, median [IQR]	4 [3–5]	3 [2–4]	4 [3–5]	0.004
Sidney Criteria, *n* (%)				
- Abortion < 10 w (≥3)	62 (66.7)	11 (45.8)	51 (73.9)	0.012
- Fetal death > 10 w	38 (40.9)	7 (29.2)	31 (44.9)	0.18
- Preterm < 34 w	10 (10.9)	6 (25)	4 (5.9)	0.018
- Thrombosis	18 (19.4)	13 (54.2)	5 (7.2)	<0.001
Obstetric morbidity, *n* (%)				
- Abortion < 10 w (≤2)	21 (22.6)	8 (33.3)	13 (18.8)	0.14
- Preterm 34–37 w	14 (15.1)	3 (12.5)	11 (15.9)	0.99
- Preeclampsia/Eclampsia > 34 w	5 (5.4)	1 (4.2)	4 (5.8)	0.99
- Abruptio Placentae	1 (1.1)	-	1 (1.4)	0.99
- IVF failures (>2)	3 (3.2)	-	3 (4.3)	0.57

IQR: interquartile range; w: weeks; IVF: in vitro fertilization failures.

**Table 3:** Main treatments in the different study groups.

	Sidney Criteria *N* = 93	2023 ACR/EULAR Criteria		
		YES *N* = 24	NO *N* = 69	*p*
Standard treatment, *n* (%)				
- LDA	86 (92.5)	21 (87.5)	65 (94.2)	0.37
- LMWH	67 (72)	21 (87.5)	46 (66.7)	0.05
- LDA + LMWH	65 (69.9)	20 (83.3)	45 (65.2)	0.09
Additional treatments, *n* (%)				
- Corticosteroids	5 (5.6)	-	5 (7.6)	0.32
- Antimalarials	8 (9.0)	3 (12.5)	5 (7.7)	0.68

LDA: low-dose aspirin; LMWH: low-molecular-weight heparin.

**Table 4:** Serological APS domains according to the 2023 ACR/EULAR classification criteria.

	Sidney Criteria *N* = 93	2023 ACR/EULAR Criteria		
		YES *N* = 24	NO *N* = 69	*p*
Domain 7, *n* (%)	38 (41.8)	14 (58.3)	24 (35.8)	0.055
- LA + persistent	34 (36.6)	14 (58.3)	20 (28.9)	0.02
Domain 8, *n* (%)	73 (78.5)	17 (70.8)	56 (81.2)	0.29
- Moderate or high IgM + (aCL and/or AB2GPI)	28 (30.1)	3 (12.5)	25 (36.2)	0.05
- Moderate IgG + (aCL+ and/or AB2GPI)	5 (5.4)	2 (8.3)	3 (4.3)	0.83
- High IgG + (aCL+ or AB2GPI)	35 (37.6)	9 (37.5)	26 (37.7)	0.82
- High IgG + (aCL+ and AB2GPI)	5 (5.4)	3 (12.5)	2 (2.9)	0.2
Combined serology, *n* (%)				
- Double+	20 (21.5)	5 (20.8)	15 (21.7)	0.93
- Triple+	11 (11.8)	5 (20.8)	6 (8.7)	0.14
- High-risk aPL profile	51 (54.8)	17 (70.8)	34 (49.3)	0.06

LA: lupus anticoagulant; aCL: anticardiolipin antibodies; AB2GPI: anti-β2 glycoprotein I.

**Table 5:** Clinical APS domains according to the 2023 ACR/EULAR classification criteria.

	Sidney Criteria *N* = 93	2023 ACR/EULAR Criteria		
		YES *N* = 24	NO *N* = 69	*p*
D1: Macrovascular (VTE), *n* (%)	12 (12.9)	9 (37.5)	3 (4.3)	<0.001
- With a high-risk VTE profile	1 (1.1)	-	1 (1.45)	0.58
- Without a high-risk VTE profile	11 (11.8)	9 (37.5)	2 (2.9)	<0.0001
D2: Macrovascular (AT), *n* (%)	6 (6.5)	4 (16.7)	2 (2.9)	0.037
- With a high-risk CVD profile	2 (2.2)	-	2 (2.9)	0.98
- Without a high-risk CVD profile	4 (4.3))	4 (16.7)	-	0.004
D3: Microvascular, *n* (%)	-	-	-	-
D4: Obstetric, *n* (%)	74 (79.6)	11 (45.8)	63 (91.3)	<0.001
- ≥3 pre-fetal Abortion (<10 w) and/or fetal (10 w 0 d–5 w 6 d) deaths	44 (47.3)	2 (8.3)	42 (60.9)	<0.0001
- Fetal (16 w 0 d–3 w 6 d) deaths in the absence of PEC or PI	20 (21.5)	1 (4.2)	19 (27.5)	0.03
- Preeclampsia with severe features (<34 w 0 d) or PI with severe features (<34 w 0 d) with/without fetal death	8 (8.6)	6 (25)	2 (2.9)	0.004
- Preeclampsia with severe features (<34 w 0 d) and PI with severe features (<34 w 0 d) with/without fetal death	2 (2.2)	2 (8.3)	-	0.11
D5: Cardiac valve, *n* (%)	-	-	-	-
D6: Thrombocytopenia, *n* (%)	12 (12.9)	11 (45.8)	1 (1.4)	<0.001

VTE: venous thromboembolism; AT: arterial thrombosis; CVD: cardiovascular disease; PI: placental insufficiency; w:weeks; d: days.

**Table 6:** Detailed APO according to the Sidney and the 2023 ACR/EULAR classification criteria.

	Sidney Criteria *N* = 93	2023 ACR/EULAR Criteria		
		YES *N* = 24	NO *N* = 69	*p*
Total APO				
- Early pregnancy loss (<10 w)	62 (66.7)	11 (45.8)	51 (73.19)	0.012
- Fetal death	38 (40.9)	7 (29.2)	31 (44.9)	0.18
- Preterm <37 w	24 (25.8)	9 (37.5)	15 (21.7)	0.13
- Preeclampsia/Eclampsia <37 w	9 (9.7)	7 (29.2)	2 (2.9)	<0.001
- Abruptio Placentae	1 (1.1)	-	1 (1.4)	0.99
APO without treatment				
- Early pregnancy loss (<10 w)	56 (65.9)	8 (44.4)	48 (71.6)	0.03
- Fetal death	30 (35.3)	5 (27.8)	25 (37.3)	0.45
- Preterm < 37 w	11 (12.9)	6 (33.3)	5 (7.5)	0.01
- Preeclampsia/Eclampsia < 37 w	7 (8.2)	5 (27.8)	2 (3.0)	0.004
- Abruptio Placentae	1 (1.1)	-	1 (1.5)	0.99
APO with treatment				
- Early pregnancy loss (<10 w)	31 (37.8)	6 (30.0)	25 (40.3)	0.41
- Fetal death	12 (14.6)	3 (15)	9 (14.5)	0.99
- Preterm < 37 w	16 (19.5)	4 (20.0)	12 (19.4)	0.99
- Preeclampsia/Eclampsia < 37 w	3 (3.7)	3 (15.0)	-	0.013
- Abruptio Placentae	-	-	-	-

APO: adverse pregnancy outcomes; w: weeks.

## Data Availability

Due to research still being conducted on the project in our research group, full data are not available. Additional data are available upon reasonable request to the corresponding author.

## Supplementary Materials

The following supporting information can be downloaded at https://www.mdpi.com/article/10.3390/diagnostics14192162/s1: Table S1: Main adverse pregnancy outcomes (APO and live birth) according to the Sidney and the ACR/EULAR 2023 classification criteria.
