# Supplementary material for: Impact of the 2023 ACR/EULAR Classification Criteria in Women with Primary Antiphospholipid Syndrome during Pregnancy
Source: Diagnostics (Basel). 2024 Sep 28;14(19):2162. doi: 10.3390/diagnostics14192162 (PMC11475906; doi:10.3390/diagnostics14192162)
Supplement: Supplementary file 1 [file diagnostics-14-02162-s001.zip › diagnostics-3200859-supplementary.pdf]

Suppl Table S1.- Main adverse pregnancy outcomes (APO and live birth) according to the Sidney and the ACR/EULAR 2023 classification criteria.

	Sidney Criteria N= 93	ACR/EULAR 2023 Criteria		
		**YES N=24**	**NO N=69**	**p**
**Total live birth**	92 (98.9)	23 (95.8)	69 (100)	0.26
- LB without treatment	34 (39.5)	11 (61.1)	23 (33.8)	0.035
- LB with treatment	64 (78.8)	19 (95.0)	45 (72.6)	0.059
**Total APO**	82 (88.8)	15 (62.5)	67 (97.1)	<0.001
- APO without treatment	74 (87.1)	13 (72.2)	61 (91.0)	0.05
- APO with treatment	51 (62.2)	10 (50.0)	41 (66.1)	0.19

LB: live birth; APO: adverse pregnancy outcomes.
